# Diverse Adiposity and Atrio-Ventricular Dysfunction across Obesity Phenotypes: Implication of Epicardial Fat Analysis

**DOI:** 10.3390/diagnostics11030408

**Published:** 2021-02-27

**Authors:** Yau-Huei Lai, Lawrence Yu-min Liu, Kuo-Tzu Sung, Jui-Peng Tsai, Wen-Hung Huang, Chun-Ho Yun, Jiun-Lu Lin, Ying-Ju Chen, Cheng-Huang Su, Ta-Chuan Hung, Chung-Lieh Hung, Jen-Yuan Kuo

**Affiliations:** 1 Department of Medicine, Mackay Medical College, New Taipei City 25245, Taiwan; garak1109@mmh.org.tw (Y.-H.L.); drlawrenceliu@gmail.com (L.Y.-m.L.); 8905012@gmail.com (K.-T.S.); 5505949.5949@mmh.org.tw (J.-P.T.); vinhank0713@msn.com (W.-H.H.); med202657@gmail.com (C.-H.Y.); jiunlulin@gmail.com (J.-L.L.); chsu007@gmail.com (C.-H.S.); jotaro3791@gmail.com (C.-L.H.); 2 Division of Cardiology, Department of Internal Medicine, Hsinchu MacKay Memorial Hospital, Hsinchu City 30071, Taiwan; 3 MacKay Junior College of Medicine, Nursing, and Management, Taipei City 11260, Taiwan; 4 Division of Cardiology, Department of Internal Medicine, MacKay Memorial Hospital, Zhongshan North Road, Taipei City 10449, Taiwan; 5 Department of Radiology, MacKay Memorial Hospital, Zhongshan North Road, Taipei City 10449, Taiwan; 6 Division of Endocrinology and Metabolism, Department of Internal Medicine, Mackay Memorial Hospital, Zhongshan North Road, Taipei City 10449, Taiwan; 7 Telehealth Center, MacKay Memorial Hospital, Zhongshan North Road, Taipei City 10449, Taiwan; ying-ju@mmh.org.tw

**Keywords:** obesity, metabolically unhealthy normal-weight, atrial fibrillation, epicardial adiposity thickness, atrio-ventricular deformations

## Abstract

Obesity has been conceptualized as a highly heterogeneous condition. We aim to investigate chamber-specific effects of obesity on the heart and relevant outcomes. A total of 2944 symptom-free individuals (age: 47.5 ± 10.0 years), free of known cardiovascular diseases were classified into four categories based on body mass index (BMI) (as normal-weight (NW) vs. overweight/obese (O)) and metabolic status (metabolically-healthy (MH) vs. unhealthy (MU)). Epicardial adipose thickness (EAT) using echocardiography method. Speckle-tracking based atrio-ventricular (LA/LV) deformations including global longitudinal strain (GLS) and peak atrial longitudinal strain (PALS) were also analyzed. MUNW had higher cardiometabolic risks and more impaired diastolic and GLS/PALS than MHNW phenotype. Both MHO and MUO phenotypes exhibited worst atrial functions. Greater EAT was independently associated with worse GLS and PALS after correcting for various anthropometrics, LV mass and LA volume, respectively, with unfavorable LA effects from EAT being more pronounced in the NW phenotypes (both *p* interactions < 0.05). During a median follow-up period of 5.3 years, BMI/EAT improved the reclassification for atrial fibrillation (AF) incidence (*p* for net reclassification improvement < 0.05) mainly in the NW phenotypes (*p* interaction < 0.001). Categorization of clinical obesity phenotypes based on excessive visceral adiposity likely provides increment prognostic impacts on atrial dysfunction, particularly in non-obese phenotypes.

## 1. Introduction

Obesity and its associated cardiometabolic disorders are well-established clinical risks for heart failure (HF) [1,2] and atrial fibrillation (AF) [3]. Along with obesity, AF is also a known risk factor for HF with preserved ejection fraction (HFpEF), an HF phenotype of emerging clinical significance [4]. To date, at least two distinct obesity phenotypes have been established: (i) an excessive body mass (defined by a large body mass index (BMI)); and (ii) an excessive ectopic fat deposition (subcutaneous or visceral adiposity). Despite the known associations between obesity and cardiac structural and functional disturbances [1,2,5,6], obesity defined by a greater BMI has been shown to be a highly heterogeneous clinical condition. Nevertheless, subjects with diagnosed HFpEF or AF may not necessarily be presented with the same degree of BMI-defined obesity [7,8,9]. 

Increasing attention has been given to individuals who are “metabolically healthy overweight/obese” (MHO), although its precise cardiometabolic risks remain controversial [10]. On the contrary, individuals with “metabolically unhealthy normal weight” (MUNW) may be subjected to adverse left ventricle (LV) remodeling over time [11]. In addition, visceral adipose tissue is proposed to be a metabolically active organ [12]. An excessive epicardial adipose tissue (EAT) has also been recognized as a distinctive HFpEF phenotype in extremely obese subjects and was shown to exert adverse biological effects on the atrium, thereby leading to a higher AF risk [13,14]. Therefore, it is possible that chamber-specific myocardial dysfunctions (atrial or ventricular) may differ among these obesity phenotypes, resulting in differential cardiovascular outcomes (i.e., AF or HF).

Speckle-tracking deformational indices using echocardiography has been demonstrated as more sensitive and relative load-independent estimates for chamber-specific myocardial metrics (atrial or ventricular) [15]. Implementing such imaging techniques would therefore serve as useful early predictors for AF or HF prior to overt structural alterations [16]. Given these associations, we aimed to investigate the potential impact of the relationships among epicardial fat, metabolic status, and evidenced subclinical myocardial dysfunction in a large-scale asymptomatic population.

## 2. Materials and Methods

### 2.1. Study Participants

We retrospectively examined individuals who participated in an ongoing cardiovascular health screening program between June 2009 and January 2013 at a tertiary medical center in Taipei, Taiwan. The original study setting and design have been published previously [5,17]. The baseline clinical information, medical history, symptoms/signs, and lifestyle patterns were obtained. Owing to the retrospective study design, informed consent was waived for each participant. This study conformed to the principles outlined in the Declaration of Helsinki and was approved by the local ethical board committee (18MMHIS180e, 8 January 2019). All study participants underwent comprehensive echocardiography assessments. To minimize potential confounders unrelated to obesity phenotypes, we excluded participants with hypertension, diabetes, and cardiovascular disease (CVD). The presence of hypertension was determined according to local practice guidelines [18] or a previous diagnosis under medication control. Diabetes was defined as a fasting plasma glucose ≥126 mg/dL and HbA1c > 6.5% on two occasions, or a previous diagnosis under medication control. CVD was defined as a history of previous myocardial infarction, symptom-driven angioplasty, peripheral arterial disease, or cerebrovascular disease. Participants with any prevalent clinical HF, significant valvular diseases, known AF, flutter, or other cardiac arrhythmias were also excluded from the analysis. Underweight subjects were also excluded.

**Figure 1:**
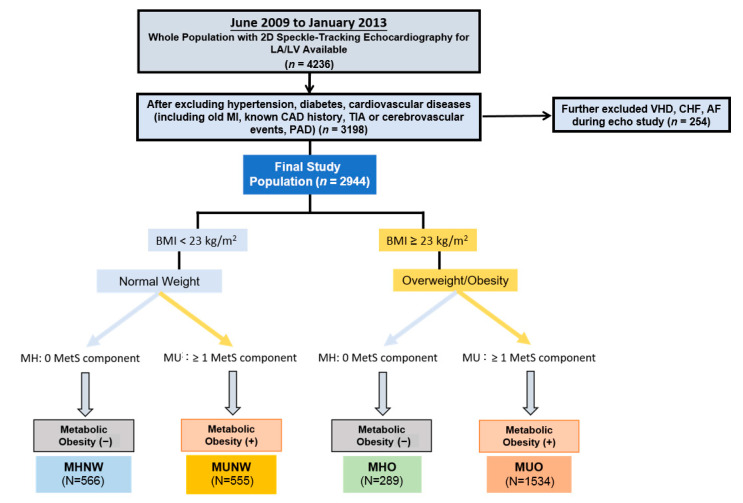
Study design and exclusion flowchart. Abbreviations: MI, myocardial infarction; CAD, coronary artery disease; TIA, transient ischemic attack; PAD, peripheral arterial disease; VHD, valvular heart disease; CHF, congestive heart failure; MetS, metabolic syndrome; MU, metabolically-unhealthy; MHNW, metabolically-healthy normal weight; MUNW, metabolically-unhealthy normal-weight; MHO, metabolically-healthy overweight/obese; MUO, metabolically-unhealthy overweight/obese.

Ultimately, 2944 participants were enrolled in the study. A more detailed flowchart is available in Figure 1.

### 2.2. Laboratory Data, Cardiac Biomarkers and HOMA-IR Analysis

Biochemical parameters, including fasting blood glucose and lipid profiles, were measured at a standardized central laboratory using a Hitachi 7170 Automatic Analyzer (Hitachi Corp., Hitachinaka, Ibaraki, Japan). N-terminal pro-brain natriuretic peptide (NT-proBNP) and high sensitivity C-reactive protein (hs-CRP) were quantified using an electrochemiluminescence immunoassay (Roche E170, Roche Diagnostics GmbH, Mannheim, Germany) and a highly sensitive, latex particle-enhanced immunoassay (Elecsys 2010; Roche Diagnostics GmbH, Mannheim, Germany), respectively. Homeostatic model assessment for insulin resistance (HOMA-IR) was calculated as: fasting blood glucose (mg/dL) × fasting insulin (µU/mL)/405. The HOMA-IR values of 1707 (57.8%) individuals were available.

### 2.3. Definition of Metabolic Health and Obesity Phenotype

The World Health Organization (WHO) expert consultation has proposed additional BMI cutoff points (23; 27.5; 32.5; 37.5 kg/m^2^) for public health action in ethnic Asians [8]. Our previous work demonstrated that Asians are susceptible to myocardial dysfunction at a much lower BMI [5]; therefore, we adopted these Asian-specific cutoffs by defining normal-weight (NW) as BMI < 23 kg/m^2^ and overweight/obese (O) as BMI ≥ 23 kg/m^2^. MHO was defined based on stricter criteria set by previous studies [19,20] as overweight or obese participants who had none of the metabolic syndrome components according to the National Cholesterol Education Program-Adult Treatment Panel III (NCEP-ATP III) [21] and the modified guidelines for ethnic Asian population [22]:

(1) Systolic blood pressure ≥ 13.0 mmHg, diastolic blood pressure ≥ 85 mmHg;
(2) Triglycerides (TG) ≥150 mg/dL;
(3) High density lipoprotein (HDL-c) <40 mg/dL in men and <50 mg/dL in women;
(4) Fasting plasma glucose ≥100 mg/dL. To align with previous studies [19,20], waist size was not included in our definition criteria due to its high collinearity with BMI.

Participants were categorized into four major obesity phenotypes, including two NW (BMI < 23 kg/m^2^) groups: metabolically healthy NW (MHNW) and MUNW; and two O (BMI ≥ 23 kg/m^2^) groups: MHO and metabolically unhealthy overweight/obese (MUO) (Figure 1).

### 2.4. Assessment of Epicardial Adipose Tissue (EAT)

Based on a previous CT-validated method [23], three repeated measures of the maximum depth at end-systole were performed at the right ventricular free wall on both para-sternal long and short-axis views to be presented as the mean EAT thickness. We further adjusted this by body surface area to be presented as the EAT index (EATi) in order to correct for body size.

### 2.5. Body Fat Composition Assessment

The body fat composition was calculated by foot-to-foot bioelectrical impedance-based analysis (BIA) (Tanita-305 Body-Fat Analyzer; Tanita Corp, Tokyo, Japan), which provides an estimate of the total body fat percentage.

### 2.6. Conventional Echocardiography and Diastolic Functional Indices

Each participant underwent an extensive two-dimensional (2D) and tissue Doppler echocardiography with strain analysis. All assessments were performed by a single experienced sonographer blinded to the participants’ clinical information, using a commercially available ultrasound system equipped with a 2–4 MHz multifrequency transducer (Vivid 7; GE Medical System, Vingmed, Norway), in adherence to the American Society of Echocardiography (ASE) guidelines [24]. LV end-diastolic/systolic and left atrium (LA) volumes (both maximal and minimal) were determined by the modified biplane Simpson’s method. All measurements were the average values derived from three consecutive cardiac cycles. Diastolic functional indices were assessed using transmitral pulsed-wave Doppler and tissue Doppler-derived mitral annular velocities. Systolic and early diastolic velocities (LV s’ and LV e’) were averaged from the basal septal and lateral LV segments at mitral annulus level. To correct for body size, we adjusted LA maximal volume by body surface area to generate the LA volume index (LAVi).

### 2.7. Two-Dimensional Speckle-Tracking Analysis Protocol

Speckle-tracking analysis was performed off-line using a 2D cardiac performance software (EchoPAC version 10.8; GE Vingmed Ultrasound, Norway). Semi-automated tracing of endocardial borders was performed at the end-diastolic frame with minor manual adjustments to ensure optimal delineation. Representative LV global longitudinal strain (GLS) was calculated as the average peak global values derived from three LV apical planes of 4-chamber, 2-chamber, and long-axis views, as described in our previously published work [5,17]. Peak atrial longitudinal strain (PALS) and triphasic LA strain rates (systolic, early, and late diastolic atrial longitudinal strain rate (ALSR_syst_, ALSR_early_, and ALSR_late_, respectively)) were determined as the average values obtained from both apical 2- and 4-chamber views. The endocardial border of the LA was traced manually so that the LA appendage and pulmonary veins were excluded. LA stiffness (LA_stiff_) was derived from dividing E/e’ by PALS. To avoid confusion regarding the directionality of strain changes, the absolute values of GLS, ALSR_early_, and ALSR_late_ were reported. The inter- and intra-observer analysis of the LA and LV strain/strain rate components in our lab were reported in our previous work [17].

### 2.8. Determination of Clinical Endpoints

After the initial data collection, we performed a chart review for all participants to evaluate their clinical outcomes. The primary endpoint of the current study was set as the adjudicated HF hospitalization and AF incidence by two cardiologists following the echocardiography study index date. Information on all endpoints was extracted from documented records in an electronic database capture system.

### 2.9. Statistical Analysis

Data were presented as means ± standard deviations for continuous variables and proportions for categorical variables. Differences in anthropometric, metabolic, and echocardiography parameters between groups were assessed by one-way ANOVA with post-hoc paired comparisons using the Bonferroni correction. A chi-squared (*χ*^2^) test was used to test differences between categorical data.

Multiple linear regression models were used to assess the cross-sectional relationships between EAT or metabolically-unhealthy (MU) conditions and key LA/LV deformations. Using MHNW as a reference group, adjusted coefficient values of the various LV/LA deformations across MUNW, MHO, and MUO were reported, and further adjusted by clinical and traditional echocardiography covariates (LV mass for models with LV deformations LAV (max) for those with LA deformations, respectively). We further examined the associations of various adiposity/anthropometric measures with abnormal LV strain (GLS < 18%), LA volume index (LAVi > 34 mL/m^2^ based on ASE guideline) [25], or LA strain (PALS < 23%) [26]. Cox regression models were conducted to test the associations of the obesity phenotypes (with MHNW as a reference group) with HF or AF events, with the same clinical covariates used for adjustments. We further examined whether the clinical utilization of EAT thresholds may provide better reclassification in identifying abnormal LV/LA strains and primary clinical endpoints. All hazard ratios are presented as the adjusted hazard ratio (aHR).

Statistical analysis was performed using STATA statistical software package (Version 12. Stata Corp. College Station, TX, USA). A two-sided *p*-value of less than 0.05 was considered statistically significant.

## 3. Results

### 3.1. Baseline Demographics, Metabolic Profiles and Adiposity Measures

Among the 2944 eligible study participants (age: 47.5 ± 10.0 years old, 66.6% males, BMI 24.1 ± 3.2kg/m^2^), both MHNW and MHO participants were younger and had better cardiometabolic profiles (i.e., lower blood pressure, lower sugar, and more favorable lipid profiles) than the MUNW participants (all *p* < 0.05) (Table 1). In general, both O phenotypes (MHO and MUO) had significantly lower NT-proBNP than both NW phenotypes (MHNW and MUNW), with both MU phenotypes (MUNW and MUO) demonstrating significantly higher HOMA-IR and hs-CRP than both MH phenotypes (MHNW and MHO) (all *p* < 0.05).

**Figure 2:**
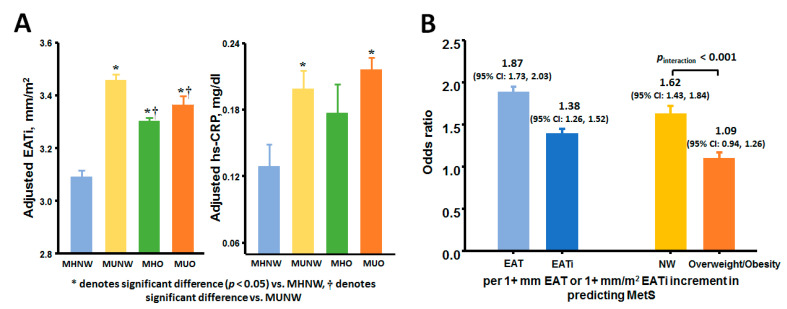
(**A**) Adjusted indexed epicardial adiposity thickness (EATi) and high sensitivity C-reactive protein (hs-CRP) across four obesity phenotypes. (**B**) Logistic regressions demonstrating the presence of metabolic syndrome per 1 mm increase in EAT (as odds ratio) and by body mass index (BMI) strata.

Overall, mean values of EAT and EATi were 6.2 ± 1.1mm and 3.3 ± 0.6mm/m^2^, respectively. Interestingly, EATi of MUNW was significantly higher than both O phenotypes (Table 2). Greater adjusted EATi coupled to higher hs-CRP levels were observed in the MU groups as compared to the MH groups (all *p* < 0.05) (Figure 2A). Increasing EAT was more closely associated with metabolic syndrome (defined as ≥ 3 components) in the NW group (adjusted coefficient: 1.62 (NW) vs. 1.09 (O), *p* interaction < 0.001) (Figure 2B). Positive linear relationships between EAT and hs-CRP and HOMA-IR were found (*r* = 0.1 and 0.24, both *p* < 0.001).

LAVi was significantly correlated with reduced PALS and increased LA stiffness (*r* = −0.2 and 0.28, both *p* < 0.001). MUNW participants had significantly worse GLS and more impaired LA mechanics overall than MHNW participants, but fared better than MHO/MUO participants (all *p* < 0.05) (Table 2). E/e’ was significantly higher in MU than in MH groups (all *p* < 0.05), although obese phenotypes exhibited a larger degree of LV/LA remodeling and worse LA strain and strain rates than NW groups. The highest LA stiffness and worst GLS were observed in MUO. The percentage of subjects with abnormal LA and LV strain (PALS < 23% and GLS < 18%) increased markedly across the obese phenotypes (both *p* < 0.001) (Table 2).

### 3.2. Associations of EAT and Metabolic Abnormalities with Atrio-Eentricular Mechanics

**Figure 3:**
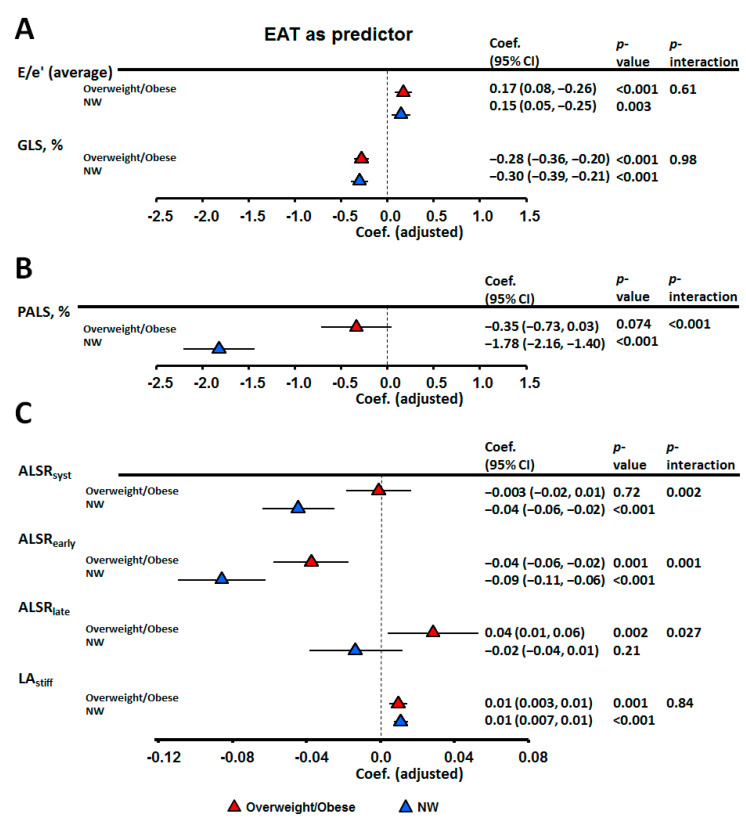
Multi-variate linear regression models exploring the relationships between epicardial adiposity thickness and various cardiac functional parameters, based on body mass index (BMI) strata. (**A**) LV indices adjusted for age, gender, BMI, systolic blood pressure (SBP), pulse rate, fasting glucose, estimated glomerular filtration rate (eGFR), triglyceride, high-density lipoprotein cholesterol (HDL-c) and left ventricle (LV) mass. (**B**,**C**) LA indices adjusted for age, gender, BMI, SBP, pulse rate, fasting glucose, eGFR, TG, HDL-c and left atrial maximum volume LAV (max).

**Figure 4:**
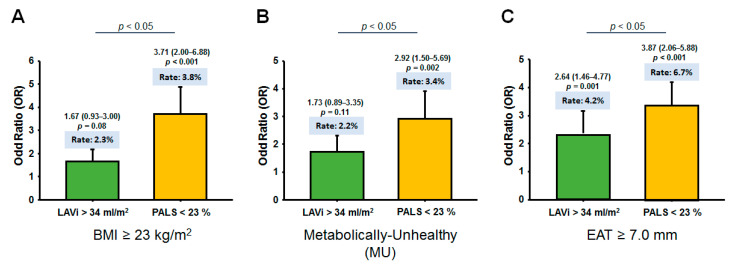
Comparisons of rates of abnormal left atrial volume index (LAVi) (>34 mL/m^2^) and abnormal left atrium (LA) strain (<23%) by various adiposity measures, defined by body mass index (BMI) strata (**A**), presence of any metabolically unhealthy (MU) condition (**B**), and excessive epicardial adiposity thickness (EAT) (≥7.0mm) (**C**).

Greater EAT was independently associated with higher E/e’, lower LV e’, GLS, and most LA mechanics (especially PALS) after correcting for clinical co-variates and LV mass (Table 3) or LAV (max), respectively (Table 4) (all *p* < 0.05). The relationships between metabolic components and myocardial functional indices were further explored (Table 5 and Table 6). Notably, associations of greater EAT with worse LA strain and strain rates were more pronounced in NW (all *p* interactions < 0.05) than in obese individuals except ALSR_late_ (Figure 3). Obese and MU phenotypes were not associated with abnormal LAVi (>34 mL/m^2^), but were significantly associated with abnormal PALS (<23%) (Figure 4). Excessive EAT (≥7.0 mm, derived from clinical outcomes) was significantly associated with both abnormal LAVi and PALS (Figure 4) even after correcting for LV mass and LAV(max) (adjusted OR (aOR): 2.64 (1.46–4.77) and 3.87 (2.06–5.88), respectively, *p* ≤ 0.001).

### 3.3. Association of Obesity Phenotypes and EAT with Clinical Outcomes

During a median follow-up period of 5.3 years (IQR: 4.5–6.3 years), HF was found in 75 subjects, whereas 71 subjects developed AF. Both MU groups had significantly more HF events (aHR = 3.2 and 5.3, respectively), while obese groups had higher AF incidence (aHR = 5.4 and 5.7, respectively) (all *p* < 0.05) (Table 7). While MU status could independently predict HF risk in fully adjusted models (aHR: 2.7, 95% confidence interval (CI): 1.1–6.4, *p* = 0.025), greater EAT remained significantly associated with AF (aHR = 1.8, 95% CI: 1.3–2.5) and HF (aHR = 1.8, 95% CI = 1.4–2.4) (both *p* < 0.001 per 1 mm EAT increase) (c-statistics: 0.71 (95% CI: 0.67–0.77) for both) incorporating BMI, LAV(max), and LVM, respectively. Compared to people with EAT < 7.0 mm, the risk for composite AF/HF events was tripled in those with EAT ≥ 7.0 mm (crude HR: 3.37 (2.18–5.22), incidence: 11.0 vs. 3.4 per 1000 person-year).

**Figure 5:**
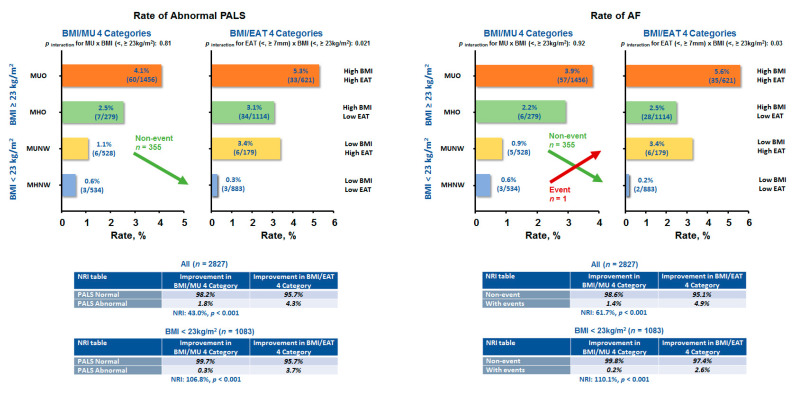
Comparison of abnormal left atrium (LA) strain and atrial fibrillation (AF) occurrence between obesity phenotypes utilizing body mass index (BMI) and metabolically unhealthy (MU) status or BMI and epicardial adiposity thickness (EAT) classification system. A large fraction of normal LA strain and non-event (AF) individuals from low BMI/MU were reclassified into the low BMI/low EAT category the in normal-weight population.

The comparisons of abnormal PALS and AF endpoint using BMI/MU and BMI/EAT for categorizing obesity phenotypes are shown in Figure 5. By utilizing a cut-off value of 7.0 mm for EAT based on a previous study [27], we observed improved reclassification of identifying abnormal PALS and higher risk for developing AF by BMI/EAT beyond BMI/MU categorization (Figure 5, all *p*-values for net reclassification improvement (NRI): <0.001), mainly in NW individuals (*p* interaction: 0.021 and 0.03, respectively). Reclassification based on BMI/EAT also showed borderline improvement beyond BMI/MU in identifying abnormal GLS (*p* for NRI: 0.065) categorizations, mainly in obese phenotypes (*p* for NRI: 0.01) (Table 8). There was no significantly improved reclassification for HF event by BMI/EAT when added to BMI/MU (*p*-value for NRI: >0.05) (Table 8).

## 4. Discussion

### 4.1. Summary of Study Results

The findings of the present study were: (i) overall, MUO subjects had the highest EAT. MUNW subjects, albeit having lower total body fats, had similarly high EAT as MHO subjects. The highest EATi was found in MUNW subjects, which paralleled to the higher insulin resistance and adjusted hs-CRP level; (ii) higher EAT was independently associated with an increase in metabolic components and impaired LA mechanics independent of LA volume, which were more pronounced in the NW groups; (iii) O phenotypes categorized using EAT and BMI improved reclassification of abnormal LA strain and AF event, especially for those with NW.

### 4.2. Utilization of Visceral Adiposity in Redefining Cardiometabolic Obesity

Over the past decade, studies have revealed that people with similar BMIs can manifest vastly heterogeneous co-morbidities and cardiovascular risks [28]. Accumulating data have suggested the pathological links between visceral fat and metabolic disorders irrespective of BMI [29], which further contributes to myocardial dysfunction and AF or HFpEF incidence [30,31]. Due to its relative ease of attainability, assessing EAT by echocardiography may be an alternative method to quantify visceral fat burden. By sharing unique and unimpeded microcirculation with adjacent myocardium, this bioactive tissue has been proposed to regulate myocardial function through the secretion of several pro-inflammatory cytokines harmful to the heart [32].

### 4.3. Effect of EAT Burden on Ventricular Remodeling and Dysfunction

The pathophysiologic basis of obesity-related diseases could be complex and multifactorial. For example, studies have shown attenuated HF risk in MHO individuals as compared to MUNW individuals [11,33]. Obese individuals had been known to have “falsely” lower natriuretic peptide levels [13], as reflected by our data. There are several hypotheses regarding this phenomenon, such as the pericardial restraint effect [13] or suppressed lipolysis [34]. Nonetheless, obesity may also confer hemodynamic benefits [35] when metabolic disorders are absent, as demonstrated by lower blood pressure and lower LV E/e’ levels in MHO compared to MUNW in the present study. These beneficial hemodynamic effects appeared to be largely reversed in MUO, leading to higher clinical events, indicating unfavorable functionality from MU condition. Despite the same amount of total body fat, MUNW subjects exhibited substantially higher EAT, higher hs-CRP/NT-proBNP levels, and worsened atrio-ventricular properties than their MHNW counterparts. Despite less body fat, MUNW subjects shared the same EAT burden as MHO subjects.

### 4.4. Effect of EAT Burden on Atrial Remodeling and Dysfunction

In comparison to obesity or metabolic abnormality, excessive EAT seemed to pose greater impacts on the atrium (Figure 4), with global LA strain as a more sensitive surrogate marker than LA volume index in delineating adiposity-related atrial myopathy [26]. AF has been proposed to be driven primarily from abnormal LA size [36], and a large extent of LA remodeling in obesity has been suggested to result from adaptive atrial remodeling influenced by systemic load status, which could explain the higher AF occurrence in such populations. Dys-regulated, excessive EAT might aggravate myocardial extracellular matrix turnover and fibrotic replacement resulting in arrhythmogenic substrate formation and AF [12,37]. Furthermore, EAT may mechanistically impede diastolic filling due to physical constraint and aggravated atrial pathology [13], as evidenced by the reduced LV e’ and LA strain/strain rates in our study (Table 3 and Table 4). Previous studies have also advocated pathophysiological links between EAT and region-specific visceral fat on LA as AF predictor irrespective of BMI [38,39].

A large, prospective HF registry revealed AF accompanied by multiple metabolic disorders to be a unique HFpEF feature in lean ethnic Asians [9]. Therefore, we speculate that excessive EAT mediating metabolic abnormalities, atrio-ventricular dysfunction and arrhythmogenic substrate formation are common in lean ethnic Asians. It is anticipated that our findings will enable the clinical implementation and utilization of EAT to better characterize cardiometabolic risks and reclassify sub-clinical LA dysfunction and AF risk than metabolic healthiness in non-obese subjects (graphical abstract). Taken collectively, these findings indicated differential biological and cardiac manifestations of diverse adiposity measures, and have demonstrated that BMI alone may be sub-optimal in characterizing true metabolic burden (Figure 2) and adiposity-related myocardial dysfunction.

### 4.5. Limitations

This study was limited by potential residual confounders attributable to its single-center retrospective analysis with post-hoc follow-up. Being a cross-sectional study, it cannot address the possible phenotypic shifts between MH and MU. Additionally, causality could not be investigated based on the availability of baseline obesity data only. Despite a non-uniform definition for MU, we adopted a relatively strict criterion to define metabolic health in order to clarify associations of purely excessive body mass with cardiac manifestations and clinical endpoints, which also resulted in a relatively large MUO group. Notably, we adopted the WHO’s BMI criteria for Asians (BMI < 23 kg/m^2^) to define NW, which resulted in relatively large O groups (graphical abstract). Finally, although we proposed excessive visceral fat as an alternative obesity phenotype with improved classification for atrial dysfunction, the cut-off derived from the present study may not be applicable to other non-Asian populations with differential cardiovascular risk profiles.

## 5. Conclusions

In conclusion, our findings have provided novel prognostic insights on the disparate cardiometabolic manifestations across diverse obesity phenotypes. We also demonstrated that visceral adiposity may play an independent role in mediating metabolic abnormality and altered atrial and ventricular mechanics. Hence, including the measures of visceral adiposity in the existing obesity phenotypes’ classification system, based on BMI and metabolic status, is anticipated to enhance atrial pathophysiology, and therefore provide a substrate for targeted therapeutic interventions.

## Figures and Tables

**Table 1:** Baseline demographics and laboratory information of study participants according to obesity phenotypes.

Total (*n* = 2944)	Normal-Weight (BMI < 23 kg/m^2^)		Overweight/Obese (BMI ≥ 23 kg/m^2^)		*p*-Value
	MHNW (*n* = 566)	MUNW (*n* = 555)	MHO (*n* = 289)	MUO (*n* = 1534)	
Demographic/Anthropometric Data					
Age, years	44.55 ± 9.88	49.25 ± 10.13 *	45.36 ± 9.09†	48.17 ± 9.81 *#	<0.001
Males (%)	217 (38.3%)	332 (59.8%)	204 (70.6%)	1209 (78.8%)	<0.001
Weight, kg	55.9 ± 7.1	58.8 ± 7.5 *	68.1 ± 7.8 *†	73.8 ± 10.7 *†#	<0.001
BMI, kg/m^2^	20.83 ± 1.26	21.45 ± 1.26 *	24.46 ± 1.62 *†	26.23 ± 2.7 *†#	<0.001
WC, cm	73.17 ± 5.55	77.4 ± 6.05 *	84.71 ± 5.59 *†	88.14 ± 8.08 *†#	<0.001
Abnormal WC (%)	36 (6.4%)	39 (7.0%)	108 (37.4%)	715 (46.6%)	<0.001
WH ratio	0.81 ± 0.06	0.85 ± 0.06 *	0.85 ± 0.05 *	0.90 ± 0.06 *†#	<0.001
WHt ratio	0.45 ± 0.03	0.46 ± 0.03 *	0.51 ± 0.04 *†	0.53 ± 0.04 *†	<0.001
BF (%)	22.77 ± 4.82	22.84 ± 5.08	24.87 ± 5.03 *†	27.91 ± 6.3 *†#	<0.001
BSA (m^2^)	1.71 ± 0.15	1.76 ± 0.15 *	1.91 ± 0.15 *†	1.99 ± 0.18 *†#	<0.001
SBP, mmHg	109.22 ± 10.55	121.2 ± 15.45 *	114.03 ± 9.58 *†	124.97 ± 15.52 *†#	<0.001
DBP, mmHg	66.9 ± 7.69	73.77 ± 10.09 *	70.57 ± 7.26 *†	77.14 ± 9.98 *†#	<0.001
Pulse rate, beats/min	71.41 ± 11.24	74.58 ± 10.84 *	70.29 ± 11.01†	74 ± 10.5 *#	<0.001
Active Smoking (%)	26 (4.6%)	51 (9.2%)	20 (6.9%)	158 (10.3%)	<0.001
Laboratory Data					
Fasting sugar, mg/dl	89.8 ± 5.86	98.72 ± 15.14 *	91.96 ± 5.23†	101.21 ± 16.79 *†#	<0.001
HOMA-IR	1.14 ± 0.6	1.54 ± 1.0 *	1.42 ± 0.79	2.25 ± 1.63 *†#	<0.001
Total cholesterol, mg/dl	197.18 ± 34.36	204.53 ± 39.63 *	201.83 ± 32.62	207.01 ± 36.61 *	<0.001
TG, mg/dl	79.95 ± 26.63	130.87 ± 103.17 *	91.56 ± 26.62 †	159.12 ± 91.09 *†#	<0.001
LDL-c, mg/dl	121.5 ± 31.63	131.8 ± 35.11 *	132.11 ± 31.8 *	137.68 ± 32.85 *†	<0.001
HDL-c, mg/dl	66.98 ± 14.82	56.17 ± 15.02 *	59.23 ± 12.1 *†	48.99 ± 12.78 *†#	<0.001
eGFR, ml/min/m^2^	94.36 ± 18.25	91.67 ± 17.82 *	89.33 ± 13.17 *	87.84 ± 15.85 *†	<0.001
Biomarkers					
NT-proBNP, pg/ml	39.56 ± 32.77	43.48 ± 70.98	30.17 ± 27.58 *†	31.36 ± 30.3 *†	<0.001
hs-CRP, mg/dl	0.10 ± 0.17	0.19 ± 0.39 *	0.16 ± 0.32	0.23 ± 0.34 *#	<0.001

*p*-value < 0.05 for comparisons against * MHNW, † MUNW, # MHO. Abbreviations: BMI, body mass index; WC, waist circumference; WH ratio, waist–hip ratio; WHt ratio, waist–height ratio; BF, body fat; BSA, body surface area; SBP, systolic blood pressure; DBP, diastolic blood pressure; HOMA-IR, homeostatic model assessment for insulin resistance; TG, triglyceride; LDL-c, low-density lipoprotein cholesterol; HDL-c, high-density lipoprotein cholesterol; eGFR, estimated glomerular filtration rate; NT-proBNP, N-terminal pro B-type natriuretic peptide; hs-CRP, high sensitivity C-reactive protein.

**Table 2:** Echocardiographic parameters of cardiac structure and mechanics according to obesity phenotypes.

Total (*n* = 2944)	Normal-Weight (BMI <23 kg/m^2^)		Overweight/Obese (BMI ≥ 23 kg/m^2^)		*p*-Value
	MHNW (*n* = 566)	MUNW (*n* = 555)	MHO (*n* = 289)	MUO (*n* = 1534)	
EAT, mm	5.35 ± 1.13	6.18 ± 1.01 *	6.25 ± 0.99 *	6.48 ± 0.93 *†#	<0.001
EATi, mm/m^2^	3.14 ± 0.70	3.53 ± 0.65 *	3.29 ± 0.56 *†	3.28 ± 0.55 *†	<0.001
LVST, mm	8.2 ± 1.1	8.5 ± 1.2 *	8.8 ± 1.1 *†	9.2 ± 1.2 *†#	<0.001
LVPT, mm	8.14 ± 1.01	8.5 ± 1.08 *	8.82 ± 1.03 *†	9.16 ± 1.12 *†#	<0.001
LVEDV, ml	66.36 ± 14.99	69.08 ± 15.5 *	79.14 ± 16.66 *†	81.44 ± 16.68 *†	<0.001
LVM, gm	117.7 ± 27.46	126.63 ± 30.44 *	142.06 ± 28.9 4*†	150.55 ± 33.13 *†#	<0.001
LVMi (Ht^2.7^), gm/m^2.7^	31.2 ± 7.15	32.73 ± 8.19 *	35.88 ± 7.54 *†	37.65 ± 9.18 *†#	<0.001
LAV(max), ml	23.56 ± 8.21	26.53 ± 9.6 *	29.3 ± 8.93 *†	34.77 ± 12.5 *†#	<0.001
LAV(min), ml	9.63 ± 4.02	10.87 ± 4.57 *	12.32 ± 4.8 *†	14.87 ± 6.35 *†#	<0.001
LAEF (%)	59.1 ± 12.61	58.39 ± 12.65	58.08 ± 11.6	57.33 ± 12.4 *	0.03
LAVi (BSA), ml/m^2^	14.4 ± 4.9	14.9 ± 5.7	15.9 ± 4.6 *	16.9 ± 6.0 *†	<0.001
LAVi > 34 mL/m^2^ (%)	6 (1.2%)	10 (1.7%)	4 (1.5%)	37 (2.4%)	0.29
DT, ms	201.8 ± 33.42	202.42 ± 32.96	201.1 ± 35.74	201.59 ± 34.13	0.83
IVRT, ms	85.19 ± 12.84	88.08 ± 12.64 *	87.85 ± 11.9 *	89.5 ± 14.25*	<0.001
E/A ratio	1.52 ± 0.44	1.3 ± 0.42 *	1.38 ± 0.44 *†	1.21 ± 0.37 *†#	<0.001
LV e’, cm/sec	11.05 ± 2.29	9.9 ± 2.22 *	10.26 ± 2.12 *	9.2 ± 2.11 *†#	<0.001
LV s’, cm/sec	8.68 ± 1.5	8.49 ± 1.49	8.49 ± 1.36	8.41 ± 1.42 *	0.005
LV E/e’ ratio	6.95 ± 1.86	7.51 ± 2.25 *	7.03 ± 1.8 †	7.75 ± 2.36 *#	<0.001
GLS (%)	21.35 ± 1.89	20.6 ± 1.74 *	20.43 ± 1.77 *	19.84 ± 1.68 *†#	<0.001
GLS < 18% (%)	15 (2.7%)	26 (4.7%)	11 (3.8%)	163 (10.6%)	<0.001
PALS (%)	42.18 ± 7.45	39.21 ± 7.54 *	37.8 ± 7.39 *	36.32 ± 7.8 *†#	<0.001
PALS < 23% (%)	3 (0.5%)	8 (1.4%)	7 (2.4%)	62 (4.0%)	<0.001
ALSR_syst_	1.85 ± 0.37	1.77 ± 0.39 *	1.64 ± 0.34 *†	1.62 ± 0.36 *†	<0.001
ALSR_early_	2.21 ± 0.51	1.94 ± 0.54 *	1.91 ± 0.48 *	1.63 ± 0.47 *†#	<0.001
ALSR_late_	1.97 ± 0.48	2.06 ± 0.47 *	1.93 ± 0.43 †	2.02 ± 0.49 #	0.001
LA_stiff_	0.17 ± 0.06	0.20 ± 0.09 *	0.20 ± 0.07 *	0.23 ± 0.11 *†#	<0.001

*p*-value < 0.05 for comparisons against * MHNW, † MUNW, # MHO groups, respectively. Abbreviations: EAT, epicardial adipose thickness; EATi, epicardial adipose thickness index; LVST, left ventricular septal wall thickness; LVPT, left ventricular posterior wall thickness; LVEDV, left ventricular end-diastolic volume; LVM, left ventricular mass; LVMi, left ventricular mass index; LAV(max), left atrial maximum volume; LAV(min), left atrial minimum volume; LAEF, left atrial emptying fraction; LAVi, left atrial volume index; DT, deceleration time; IVRT, isovolumetric relaxation time; E/A, early-to-late diastolic mitral inflow velocity ratio; e’, early-diastolic tissue Doppler velocity; s’, systolic tissue Doppler velocity; GLS, left ventricular global longitudinal strain; PALS, peak atrial longitudinal strain; ALSR_syst_, atrial longitudinal strain rate-systolic phase; ALSR_early_, atrial longitudinal strain rate-early diastolic phase; ALSR_late_, atrial longitudinal strain rate-late diastolic phase; LA_stiff_, left atrial stiffness.

**Table 3:** Multivariate regression analysis of epicardial adipose tissue thickness (EAT) with ventricular structure and mechanics.

LV Indices		LV s’			LV e’			E/e’			GLS		
Pearson’s *R*		−0.08			−0.27			0.14			−0.27		
Regression models		Coef.	95% CI	*p*	Coef.	95% CI	*p*	Coef.	95% CI	*p*	Coef.	95% CI	*p*
Uni-variate		−0.11	−0.16; −0.06	<0.001	−0.58	−0.66; −0.5	<0.001	0.3	0.22; 0.37	<0.001	−0.47	−0.53; −0.41	<0.001
Multi-variate													
Model 1 (+ Age)		−0.02	−0.07; 0.03	0.44	−0.33	−0.39; −0.26	<0.001	0.13	0.06; 0.2	<0.001	−0.46	−0.53; −0.4	<0.001
BMI-based	Age + BMI	0.01	−0.04; 0.06	0.73	−0.15	−0.22; −0.09	<0.001	0.08	0.003; 0.16	0.04	−0.3	−0.36; −0.23	<0.001
	Age + BMI + CV	−0.003	−0.05; 0.05	0.92	−0.13	−0.19; −0.06	<0.001	0.09	0.02; 0.17	0.02	−0.25	−0.32; −0.19	<0.001
	Age + BMI + CV + LVM	0.01	−0.04; 0.06	0.79	−0.12	−0.18; −0.05	0.001	0.08	0.01; 0.16	0.03	−0.25	−0.31; −0.19	<0.001
WC-based	Age + WC	−0.01	−0.07; 0.04	0.61	−0.17	−0.23; −0.1	<0.001	0.12	0.05; 0.2	0.002	−0.28	−0.35; −0.22	<0.001
	Age + WC + CV	−0.01	−0.06; 0.04	0.72	−0.14	−0.2; −0.07	<0.001	0.1	0.03; 0.18	0.007	−0.26	−0.32; −0.2	<0.001
	Age + WC + CV + LVM	0.004	−0.05; 0.06	0.88	−0.12	−0.19; −0.06	<0.001	0.09	0.01; 0.16	0.02	−0.25	−0.32; −0.19	<0.001
BF-based	Age + BF	0.03	−0.03; 0.08	0.33	−0.27	−0.33; −0.2	<0.001	0.06	−0.02; 0.13	0.13	−0.45	−0.51; −0.38	<0.001
	Age + BF + CV	−0.01	−0.06; 0.05	0.84	−0.13	−0.19; −0.06	<0.001	0.11	0.03; 0.18	0.005	−0.24	−0.31; −0.18	<0.001
	Age + BF + CV + LVM	0.01	−0.04,0.06	0.74	−0.11	−0.18; −0.04	0.001	0.09	0.02,0.17	0.02	−0.24	−0.3; −0.17	<0.001

Abbreviations same as Table 1 and Table 2. CV (covariates): sex, systolic pressure, pulse rate, fasting glucose, eGFR, TG, HDL-c.

**Table 4:** Multivariate regression analysis of epicardial adipose tissue thickness (EAT) with atrial structure and mechanics.

LA Indices		PALS			ALSR_syst_			ALSR_early_			ALSR_late_			LA_stiff_		
Pearson’s *R*		−0.26			−0.17			−0.33			0.04			0.24		
Regression models		Coef.	95% CI	*p*	Coef.	95% CI	*p*	Coef.	95% CI	*p*	Coef.	95% CI	*p*	Coef.	95% CI	*p*
Uni-variate		−1.99	−2.26; −1.72	<0.001	−0.06	−0.07; −0.05	<0.001	−0.17	−0.18; −0.15	<0.001	0.02	0.003; 0.04	0.02	0.022	0.018,0.025	<0.001
Multi-variate																
Model 1 (+Age)		−1.68	−1.95; −1.4	<0.001	−0.05	−0.06; −0.04	<0.001	−0.11	−0.13; −0.1	<0.001	0.01	−0.01; 0.03	0.31	0.015	0.011; 0.018	<0.001
BMI-based	Age + BMI	−1.05	−1.33; −0.76	<0.001	−0.02	−0.03; −0.004	0.01	−0.05	−0.07; −0.03	<0.001	0.01	−0.01; 0.03	0.21	0.009	0.006; 0.012	<0.001
	Age + BMI + CV	−0.98	−1.27; −0.7	<0.001	−0.02	−0.03; −0.01	0.005	−0.04	−0.06; −0.03	<0.001	0.005	−0.01; 0.02	0.63	0.009	0.006; 0.012	<0.001
	Age + BMI + CV + LAV(max)	−0.88	−1.16; −0.59	<0.001	−0.01	−0.03; −0.001	0.04	−0.04	−0.06; −0.03	<0.001	0.01	−0.01; 0.03	0.21	0.007	0.004; 0.01	<0.001
WC-based	Age + WC	−1.13	−1.41; −0.85	<0.001	−0.03	−0.04; −0.02	<0.001	−0.06	−0.08; −0.04	<0.001	0.004	−0.01; 0.02	0.69	0.011	0.008; 0.014	<0.001
	Age + WC + CV	−1.05	−1.33; −0.77	<0.001	−0.03	−0.04; −0.01	<0.001	−0.05	−0.07; −0.04	<0.001	0.001	−0.02; 0.02	0.9	0.01	0.007; 0.013	<0.001
	Age + WC + CV + LAV(max)	−0.91	−1.19; −0.63	<0.001	−0.02	−0.03; −0.01	0.006	−0.05	−0.06; −0.03	<0.001	0.01	−0.01; 0.03	0.28	0.007	0.004; 0.011	<0.001
BF-based	Age + BF	−1.45	−1.72; −1.17	<0.001	−0.03	−0.05; −0.02	<0.001	−0.09	−0.11; −0.08	<0.001	0.02	0.01; 0.04	0.008	0.01	0.007; 0.013	<0.001
	Age + BF + CV	−1.02	−1.3; −0.73	<0.001	−0.02	−0.04; −0.01	0.001	−0.05	−0.07; −0.04	<0.001	0.01	−0.01; 0.02	0.53	0.009	0.005; 0.012	<0.001
	Age + BF + CV + LAV(max)	−0.88	−1.17; −0.59	<0.001	−0.02	−0.03; −0.002	0.02	−0.05	−0.06; −0.03	<0.001	0.01	−0.01; 0.03	0.16	0.006	0.003; 0.01	<0.001

Abbreviations same as Table 1 and Table 2. CV (covariates): sex, systolic pressure, pulse rate, fasting glucose, TG, HDL-c, and eGFR.

**Table 5:** Multivariate linear regression analysis on associations of metabolic components or epicardial fat thickness (EAT) with LV diastolic/deformation parameters as dependent variables.

Models	Univariate Model		Multivariate Models							
			Multivariate Model 1		Multivariate Model 2		Multivariate Model 3		Multivariate Model 4	
**LV e’**	**Coef.**	***p***	**Coef.**	***p***	**Coef.**	***p***	**Coef.**	***p***	**Coef.**	***p***
BP Abnormality	−1.18	<0.001	−1.03	<0.001	−0.9	<0.001	−0.49	<0.001	−0.36	<0.001
Sugar Abnormality	−0.83	<0.001	−0.58	<0.001	−0.45	<0.001	−0.07	0.37	−0.02	0.77
TG Abnormality	−0.91	<0.001	−0.7	<0.001	−0.57	<0.001	−0.66	<0.001	−0.63	<0.001
HDL Abnormality	−0.46	<0.001	−0.19	0.1	−0.1	0.37	−0.2	0.03	−0.13	0.14
EAT	−0.58	<0.001	—	—	−0.45	<0.001	−0.22	<0.001	−0.18	<0.001
**E/e’**	**Coef.**	***p***	**Coef.**	***p***	**Coef.**	***p***	**Coef.**	***p***	**Coef.**	***p***
BP Abnormality	0.67	<0.001	0.64	<0.001	0.57	<0.001	0.45	<0.001	0.39	<0.001
Sugar Abnormality	0.34	<0.001	0.23	0.02	0.16	0.09	0.06	0.5	0.06	0.5
TG Abnormality	0.17	0.08	−0.03	0.8	−0.1	0.34	0.25	0.008	0.26	0.005
HDL Abnormality	0.39	<0.001	0.39	0.001	0.34	0.002	0.3	0.003	0.25	0.02
EAT	0.3	<0.001	—	—	0.24	<0.001	0.15	<0.001	0.12	0.001
**GLS, %**	**Coef.**	***p***	**Coef.**	***p***	**Coef.**	***p***	**Coef.**	***p***	**Coef.**	***p***
BP Abnormality	−0.72	<0.001	−0.62	<0.001	−0.5	<0.001	−0.37	<0.001	−0.27	0.01
Sugar Abnormality	−0.54	<0.001	−0.35	<0.001	−0.25	0.001	−0.11	0.15	−0.07	0.38
TG Abnormality	−0.84	<0.001	−0.68	<0.001	−0.57	<0.001	−0.32	<0.001	−0.29	<0.001
HDL Abnormality	−0.5	<0.001	−0.23	0.01	−0.16	0.08	−0.25	0.005	−0.21	0.02
EAT	−0.47	<0.001	—	—	−0.38	<0.001	−0.32	<0.001	−0.3	<0.001

Model 1: all MetS components weighed together in the same model; Model 2: Model 1 further adjusted for EAT; Model 3: Model 2 adjusted for age and gender; Model 4: Model 2 adjusted for age, gender, heart rate, eGFR, and LV mass. Abbreviations same as Table 1 and Table 2.

**Table 6:** Multivariate linear regression analysis on associations of metabolic components or epicardial fat thickness (EAT) with LA diastolic/deformation parameters as dependent variables.

Models	Univariate Model		Multivariate Models							
			Multivariate Model 1		Multivariate Model 2		Multivariate Model 3		Multivariate Model 4	
**PALS, %**	**Coef.**	***p***	**Coef.**	***p***	**Coef.**	***p***	**Coef.**	***p***	**Coef.**	***p***
**BP Abnormality**	−2.34	<0.001	−1.98	<0.001	−1.47	<0.001	−0.91	0.006	−0.86	0.01
**Sugar Abnormality**	−1.88	<0.001	−1.28	<0.001	−0.8	0.02	−0.28	0.4	−0.21	0.53
**TG Abnormality**	−2.59	<0.001	−2.08	<0.001	−1.58	<0.001	−1.55	<0.001	−1.48	<0.001
**HDL Abnormality**	−1.48	<0.001	−0.65	0.12	−0.3	0.45	−0.47	0.23	−0.36	0.37
**EAT**	−1.99	<0.001	—	—	−1.72	<0.001	−1.42	<0.001	−1.21	<0.001
**ALSR_early_**	**Coef.**	***p***	**Coef.**	***p***	**Coef.**	***p***	**Coef.**	***p***	**Coef.**	***p***
**BP Abnormality**	−0.24	<0.001	−0.24	<0.001	−0.2	<0.001	−0.12	<0.001	−0.1	<0.001
**Sugar Abnormality**	−0.24	<0.001	−0.19	<0.001	−0.15	<0.001	−0.08	<0.001	−0.07	<0.001
**TG Abnormality**	−0.22	<0.001	−0.16	<0.001	−0.13	<0.001	−0.14	<0.001	−0.12	<0.001
**HDL Abnormality**	−0.15	<0.001	−0.08	0.003	−0.05	0.04	−0.08	0.001	−0.06	0.006
**EAT**	−0.16	<0.001	—	—	−0.13	<0.001	−0.09	<0.001	−0.07	<0.001
**LA_stiff_**	**Coef.**	***p***	**Coef.**	***p***	**Coef.**	***p***	**Coef.**	***p***	**Coef.**	***p***
**BP Abnormality**	0.03	<0.001	0.03	<0.001	0.02	<0.001	0.02	<0.001	0.01	0.001
**Sugar Abnormality**	0.02	<0.001	0.02	<0.001	0.01	0.005	0.005	0.23	0.004	0.23
**TG Abnormality**	0.02	<0.001	0.01	0.07	0.003	0.54	0.01	0.002	0.01	0.006
**HDL Abnormality**	0.02	<0.001	0.02	<0.001	0.01	0.003	0.01	0.001	0.01	0.004
**EAT**	0.02	<0.001	—	—	0.02	<0.001	0.01	<0.001	0.01	<0.001

Model 1: all MetS components weighed together in the same model; Model 2: Model 1 further adjusted for EAT; Model 3: Model 2 adjusted for age and gender; Model 4: Model 2 adjusted for age, gender, heart rate, eGFR, and LA volume. Abbreviations same as Table 1 and Table 2.

**Table 7:** Cox regression analysis for cardiovascular events according to obesity phenotypes (total *n* = 2944).

Event Types	AF Event				HF Event			
**Obesity Phenotypes by BMI vs. Metabolic status**	**Rate (%)**	**aHR**	**(95% CI)**	***p*-value**	**Rate (%)**	**aHR**	**(95% CI)**	***p*-value**
Low BMI/metabolically healthy (MHNW)	0.4	1	(reference)	—	0.6	1	(reference)	—
Low BMI/metabolically unhealthy (MUNW)	1.1	1.3	(0.2–7.0)	0.75	2.1	3.2	(1.0–11.9)	0.04
High BMI/metabolically healthy (MHO)	2.3	5.4	(1.2–31.5)	0.04	0.8	1.8	(0.4–9.3)	0.46
High BMI/metabolically unhealthy (MUO)	3.8	5.7	(1.7–30.6)	0.02	3.6	5.3	(1.5–18.5)	0.01
**Obesity Phenotypes by BMI vs. Visceral Fat**	**Rate (%)**	**aHR**	**(95% CI)**	***p*-value**	**Rate (%)**	**aHR**	**(95% CI)**	***p*-value**
Low BMI/low EAT (<7.0 mm)	0.2	1	(reference)	—	0.8	1	(reference)	—
Low BMI/high EAT (≥7.0 mm)	3.2	9.3	(1.8−48.1)	0.008	4.3	3.6	(1.2−10.4)	0.018
High BMI/low EAT (<7.0 mm)	2.5	7.4	(1.7–31.7)	0.007	2.2	2.3	(0.9–5.7)	0.065
High BMI/high EAT (≥7.0 mm)	5.6	13.8	(3.3–59.9)	<0.001	4.8	4.2	(1.7–10.2)	0.002

Abbreviations same as Table 1. aHR, adjusted hazard ratio. Models adjusted for clinical covariates including age, sex, systolic pressure, pulse rate, fasting glucose, triglyceride, HDL-c, eGFR plus LAV(max) for AF; and adjusted for clinical covariates plus LVM for HF. There were 71 AF events and 75 HF events with data from both categorizations available.

**Table 8:** Reclassification of abnormal LV strain (GLS) and HF event based on BMI/MU and BMI/EAT categories.

**NRI table for abnormal GLS (<18%)**		
**All, *n* = 2827**	**Improvement in BMI/MU 4 Category**	**Improvement in BMI/EAT 4 Category**
GLS Normal	93.7%	91.1%
GLS Abnormal	6.3%	8.1%
NRI: 13.2%, *p* = 0.065		
**BMI ≥ 23 kg/m^2^, *n* = 1744**	**Improvement in BMI/MU 4 Category**	**Improvement in BMI/EAT 4 Category**
GLS Normal	92.1%	88.7%
GLS Abnormal	7.9%	11.3%
NRI: 19.9%, *p* = 0.01		
**NRI table for HF event**		
**All, *n* = 2827**	**Improvement in BMI/MU 4 Category**	**Improvement in BMI/EAT 4 Category**
Non-event	97.8%	97.2%
With events	2.2%	2.8%
NRI: 12.5%, *p* = 0.29		
**BMI ≥ 23 kg/m^2^, *n* = 1744**	**Improvement in BMI/MU 4 Category**	**Improvement in BMI/EAT 4 Category**
Non-event	97.3%	96.1%
With events	2.7%	3.9%
NRI: 19.9%, *p* = 0.15		

## Data Availability

Owing to local institutional regulation (which in this study was approved years ago and at that stage the authors did not apply for data spread or distribution out of the institution), together with the newly applied “Personal Information Protection Act" in Taiwan, the data will not be appropriate to be released in public place. The spread and data release will cause some concern from local ethical committee based on current institution regulations. Data are available from the "MacKay Memorial Hospital” Institutional Data Access/Ethics Committee for researchers who meet the criteria for access to confidential data. The contact information as follows: Mackay Memorial Hospital, Address: No. 92, Sec. 2, Zhongshan N. Rd., Taipei City 10449, Taiwan, Tel: 02-25433535#3486~3488, Email: mmhirb82@gmail.com (Institutional Review Board).
